# Comparison of mucin levels at the ocular surface of visual display terminal users with and without dry eye disease

**DOI:** 10.1186/s12886-023-02931-3

**Published:** 2023-04-28

**Authors:** Hongyu Duan, Tingting Yang, Yifan Zhou, Baikai Ma, Lu Zhao, Jiawei Chen, Hong Qi

**Affiliations:** 1 Department of Ophthalmology, Peking University Third Hospital, Beijing Key Laboratory of Restoration of Damaged Ocular Nerve, 49 North Garden Rd, Haidian District, Beijing, 100191 China; 2 Institute of Medical Technology, Peking University Health Science Center, Beijing, China; 3 Department of Ophthalmology, Guangdong Provincial People’s Hospital, Guangdong Academy of Medical Sciences, Guangzhou, China

**Keywords:** Dry eye disease, Membrane-associated mucins, Visual display terminal

## Abstract

**Background:**

The long-term use of visual display terminals (VDT) is linked to an increased risk of dry eye disease (DED). Numerous studies have indicated that ocular mucins play a vital role in the pathogenesis of DED. Therefore, we aimed to evaluate (1) whether mRNA levels of membrane-associated mucins (MAMs), including MUC1, MUC4, MUC16, and MUC20, as well as MUC5AC are altered in conjunctival cells of VDT users with and without DED and (2) the relationship between mucin levels and subjective and objective tests of DED in VDT users.

**Methods:**

Seventy-nine VDT users were enrolled and divided into DED (n = 53) and control (n = 26) groups. All participants were evaluated for parameters of DED using the Ocular Surface Disease Index (OSDI) questionnaire, tear breakup time (TBUT), corneal fluorescein staining (CFS), lissamine green (LG) staining, and tear meniscus height (TMH). Based on the conjunctival impression cytology (CIC) method, differences in MUC1, MUC4, MUC16, MUC20, and MUC5AC mRNA expression levels were observed between the DED and control groups, and between symptomatic and asymptomatic participants.

**Results:**

The DED group showed significantly decreased MUC1, MUC16, and MUC20 expressions (all P < 0.05) compared to the control group. In addition, these mucin levels were lower in subjects with frequent ocular symptoms (foreign body sensation, blurred vision and painful or sore eyes) than in asymptomatic participants (all P < 0.05). Correlation analysis revealed that MUC1, MUC16, and MUC20 levels in VDT users were positively correlated with TBUT or TMH, or both. However, no significant relationship was found between MUC4 and MUC5AC levels and the DED parameters.

**Conclusion:**

VDT users with an increased frequency of ocular discomfort or a diagnosis of DED had a decreased MUC1, MUC16 and MUC20 mRNA expression in their conjunctival cells. MAMs deficiency in the conjunctival epithelium may be one of the mechanisms leading to tear film instability and DED in VDT users.

## Introduction

Dry eye disease (DED) is a multifactorial disorder characterised by loss of homeostasis of the tear film. It is accompanied by various symptoms, including ocular surface dryness, stinging, burning, pain, and foreign body sensation [1]. The estimated prevalence of DED ranges from 5 to 50% depending on the study population and diagnostic criteria [2]. According to the the Tear Film and Ocular Surface Society (TFOS) Dry Eye Workshop (DEWS) II reports, identified risk factors for DED include intrinsic factors; such as ageing, the female sex, ocular diseases, and certain systemic and autoimmune diseases; as well as extrinsic risk factors, including androgen deficiency, computer use, contact lens wear, and so on.

In recent years, the number of people using visual display terminals (VDTs), including computers, smartphones, and tablets, has increased dramatically. Global Internet users accounted for 17% of the total in 2005. By 2019, this percentage had more than tripled to 51% [3]. The ongoing COVID-19 pandemic has further accelerated people’s reliance on VDT use even more [4]. The long-term use of VDT is linked to an increased risk of DED, and the estimated global prevalence of DED in VDT users ranges from 26–70% [5–7]. The prevailing view is that, VDT use reduces the blink rate and increases the proportion of incomplete blinks, resulting in increased exposure of the ocular surface to the environment and excessive tear evaporation, ultimately leading to a vicious cycle of DED [8–10]. In addition, most VDT-associated DED belongs to the short tear breakup time DED, mainly characterised by tear film instability, and mucins play a major role in its development [5, 11]. However, the exact mechanisms leading to DED development following VDT use have not been elucidated.

Mucins are large and complex heavily glycosylated proteins, which are vital for protecting and maintaining the integrity of the ocular surface. Nearly 20 mucin genes have been identified in humans, and their products are divided into two categories: membrane-associated mucins (MAMs) and secreted mucins. Several MAMs, including MUC1, MUC4, MUC16, and MUC20, have been identified at the ocular surface in mRNA or protein levels, or both [12–15]. The most important and prevalent secreted mucin at the ocular surface is MUC5AC, which is secreted by the conjunctival goblet cells. Numerous studies have indicated that alterations of ocular mucins are intermediate links in the pathogenesis of DED as well as the consequence of DED [16]. However, there is still a lack of consistency regarding the alteration of mucins in DED because no distinction has been made between different types of DED. In addition, researchers have reported that MUC5AC concentrations were lower in the tears of VDT users with DED [17]. Nonetheless, it is unknown whether other MAMs levels are altered in patients with VDT-associated DED, and the relationship between mucin changes and the functional consequences of DED.

In the present study, we compared the mRNA levels of MUC1, MUC4, MUC16, MUC20, and MUC5AC in conjunctival impression cytology (CIC) samples from VDT users with DED to those without DED. Furthermore, we explored the relationship between mucin expression levels and (1) the frequency of ocular symptoms, and (2) the parameters of DED.

## Materials and methods

### Participants

This cross-sectional study recruited subjects by advertisement through online resources and posters at the Peking University Health Science Center campus from March 2021 to December 2021. Participants who were exposed to VDTs for more than six months (at least 5 days per week and 6 h per day) were enrolled and divided into two groups: (1) VDT users with DED and (2) VDT users without DED. DED was diagnosed according to TFOS DEWS II criteria: (1) Ocular Surface Disease Index (OSDI) score ≥ 13 and (2) one of these signs: tear breakup time (TBUT) < 10 s; abnormal ocular surface staining (> 5 corneal spots or > 9 conjunctival spots) [1]. This study was approved by the Research Ethics Committee of Peking University Third Hospital (registration number: M2020402) and performed in accordance with the Declaration of Helsinki. Informed consent was obtained from each participant prior to the study.

The exclusion criteria were as follows: a history of contact lens wear or ocular surgery within 2 years of the study visit, topical or systemic therapies other than artificial tears within two weeks before recruitment, inflammatory ocular diseases, ocular surface diseases, glaucoma, and systemic diseases with ocular involvement, such as meibomian gland dysfunction, diabetes, and peripheral neuropathy.

### Ocular clinical measurements

Eye with heavier dry eye signs was selected for evaluation and statistical analysis. The subjective symptoms of DED were assessed using the 12-item OSDI questionnaire. It includes five ocular symptom items (*sensitivity to light*, *foreign body sensation*, *painful or sore eyes*, *blurred vision* and *poor vision*), four daily activity items, and three environmental trigger items. Possible answers to each item included 4 (*all of the time*), 3 (*most of the time*), 2 (*half of the time*), 1 (*some of the time*), and 0 (*none of the time*). The participants were divided into two groups based on their answers to the five ocular symptom items: symptomatic group (item score ≥ 3) and asymptomatic group (item score ≤ 2). After applying a moist fluorescein strip to the inferior fornix, TBUT was measured by calculating the average of three consecutive break-up times, and corneal fluorescein staining (CFS) was performed based on the National Eye Institute grading system. Conjunctival staining was measured using lissamine green (LG) staining following the Oxford Scheme. Tear meniscus height (TMH) was evaluated using a Keratograph 5 M noninvasive ocular surface analyser (Oculus, Germany).

### Collection of conjunctival impression cytology

A drop of topical anaesthetic 0.4% oxybuprocaine hydrochloride (Benoxil, Santen, Japan) was applied to the eye. After for 1 min, a sterilised nitrocellulose membrane (Millipore, USA, size: 5 × 5 mm^2^) was placed on the temporal bulbar conjunctiva adjacent to the corneal limbus and pressed gently against with forceps for 5–10 s [18]. The imprint was then removed from the eye, transferred into an Eppendorf tube containing RLT *lysis buffer* (Qiagen, CA, USA) supplemented with 0.1% *β*-mercaptoethanol (Sigma-Aldrich), and stored at -80 °C until the time of extraction. One drop of topical ophthalmic antibiotic formulation was applied to the ocular surface following the collection.

### RNA extraction and reverse transcription

Total RNA was extracted using an extraction kit (RNeasy Mini Kit; Qiagen) according to the manufacturer’s instructions. Then cDNA was synthesised from total RNA with the RevertAid First Strand cDNA Synthesis Kit (K1622, Thermo Scientific). Reverse transcription was performed at 42 °C for 60 min, followed by inactivation at 70 °C for 5 min. Reverse transcription products were prepared for real-time polymerase chain reaction (PCR).

### Quantitative real-time PCR

Real-time PCR was performed using SybrGreen Master Mix and a 7500 Real-Time PCR System (Applied Biosystems, Carlsbad, CA, USA). All reactions were run in three replicates for each sample. The following thermocycling conditions were used under the standard mode according to the manufacturer’s recommendations: 30 s at 95 °C followed by 40 cycles of 95 °C for 5 s and 60 °C for 34 s. Relative mRNA expression was determined by comparing the threshold cycle of amplified genes (MUC1, MUC4, MUC16, MUC20, and MUC5AC) with that of glyceraldehyde-3-phosphate dehydrogenase (GAPDH) using the ΔCT method. The sequences of the PCR primers are listed in Table 1.

**Table 1:** Primer sequences for real-time PCR.

Gene	Forward Primer (5′-3′)	Reverse Primer (5′-3′)	PCR product, bp
GAPDH	GAAGGTGAAGGTCGGAGTC	GGAAGATGGTGATGGGATTT	227
MUC1	GTGCCCCCTAGCAGTACCG	GACGTGCCCCTACAAGTTGG	123
MUC4	GCCCAAGCTACAGTGTGACTCA	ATGGTGCCGTTGTAATTTGTTGT	102
MUC16	AGTGTCCTTGTGGATGGGTA	GATCCTCCAGGTCTAGGTGT	229
MUC20	CCTCACTTCCAGGTCTCCTT	CCTCTCAGCACAGTAACGCA	146
MUC5AC	CGACCTGTGCTGTGTACCAT	GTGCAGGGTCACATTCCTCA	197

### Statistical analysis

Statistical analysis was performed using SPSS Version 23.0 (SPSS Inc., Chicago, IL, United States). Continuous variables were expressed as mean (standard deviation) or median (interquartile range) according to their distributions. Categorical data were presented as numbers. Independent samples t-test or Mann–Whitney U test was used to compare the DED and control groups when data was normally or non-normally distributed, respectively. The Chi-square test was used to compare qualitative data between the two groups. Mucin levels in the symptomatic and asymptomatic groups were compared using the Mann–Whitney U test. Spearman’s rank correlation was applied to explore the relationship between mucin levels and parameters of DED. Statistical significance was set at P < 0.05.

## Results

### Demographics and ocular surface parameters

The study included 79 eyes of 79 VDT users, with 53 eyes of 53 individuals in the DED group and 26 eyes of 26 individuals in the control group. Demographic data and ocular surface parameters are shown in Table 2. No significant difference was observed between the two groups regarding age, sex, spherical equivalent (SE), or VDT use time (P = 0.172, 0.621, 0.991, and 0.052, respectively). Compared to the control group, the DED group had higher OSDI, CFS, and LG scores (all P < 0.05), but shorter TBUT and TMH (both P < 0.05).

**Table 2:** Demographics and ocular surface parameters of subjects

Characteristics*	DED	Control	*P*-value
Subjects/eyes, n/n	53/53	26/26	-
Age, yr	23.66 (2.95)	22.77 (2.10)	0.172^†^
Female/Male, n/n	38/15	20/6	0.621^‡^
SE, D	-4.78 (3.29)	-4.77 (1.55)	0.991^†^
VDT use time, h/d	8.08 (1.86)	8.88 (1.20)	0.052^†^
Ocular surface parameters			
OSDI score	41.67 (32.46)	19.10 (17.99)	< 0.001^§^*
TBUT, s	3.00 (2.00)	12.00 (5.00)	< 0.001^§^*
CFS score	1.00 (3.00)	0.00 (0.00)	0.002^§^*
LG score	0.00 (1.00)	0.00 (0.00)	0.001^§^*
TMH, mm	0.20 (0.06)	0.24 (0.06)	0.011^†^*

Continuous variables are displayed as mean (standard deviation) or median (interquartile range) according to their distributions. Categorical data are displayed as numbers. ^†^Independent samples t-test. ^‡^χ^2^ test. ^§^Mann-Whitney U test. SE, spherical equivalent. OSDI, Ocular Surface Disease Index. TBUT, tear breakup time. CFS, corneal fluorescein staining. LG, lissamine green staining. TMH, tear meniscus height. *P < 0.05 between groups

### Mucin levels between DED and control group

**Fig. 1:**
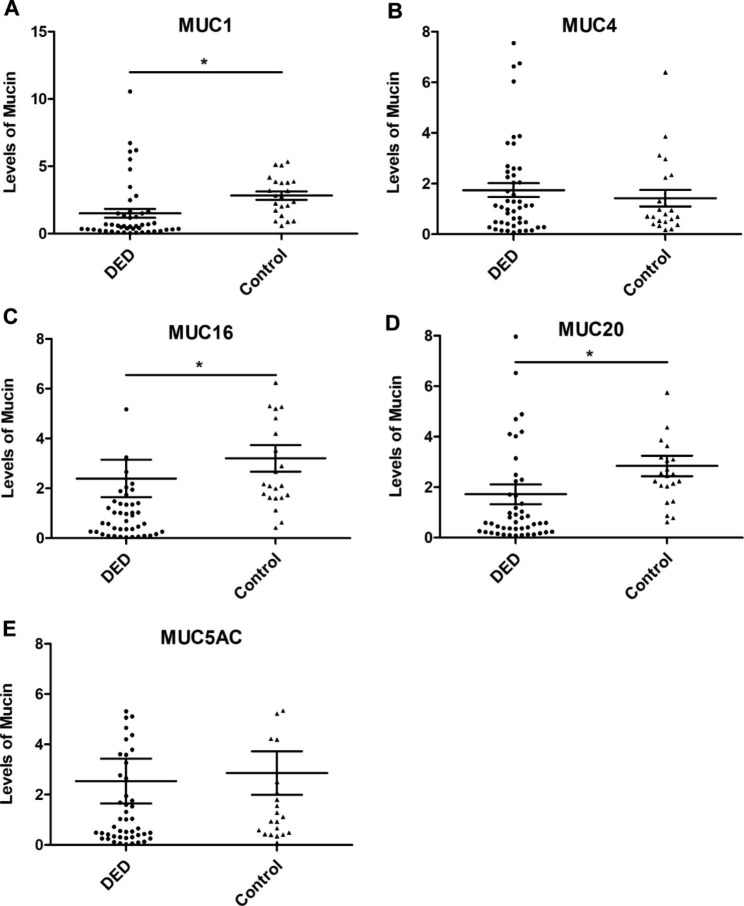
Comparisons of mucins in VDT users with and without DED. The mRNA levels of MUC1 (**A**), MUC4 (**B**), MUC20 (**C**), MUC16 (**D**), and MUC5AC (**E**) in conjunctival cells of participants in DED and control group. *P < 0.05 between groups

Between-group comparisons of MUC1, MUC4, MUC16, MUC20, and MUC5AC levels are shown in Fig. 1. The DED group showed lower levels of MUC1, MUC16, and MUC20 than those in the control group (all P < 0.001). There were no significant differences in MUC4 and MUC5AC levels between the DED and control groups (P = 0.748 and P = 0.226, respectively).

### Relationship between mucin expression and ocular symptom frequency

The relationships between MUC1, MUC4, MUC16, MUC20, and MUC5AC expression levels and ocular symptom frequency are shown in Table 3. Based on our classification of DED symptoms frequency, we found that MUC1, MUC16, and MUC20 expression were lower in the symptomatic group than in the asymptomatic group on foreign body sensation (P = 0.043, 0.017 and 0.007, respectively). For painful or sore eyes, the symptomatic group showed decreased MUC20 expression compared with the asymptomatic group (P = 0.031). Regarding blurred vision, the symptomatic group showed lower MUC1, MUC16, and MUC20 expression levels than the asymptomatic group (P = 0.011, 0.023, and 0.009, respectively). There were no significant differences between the five mucin expressions and the frequencies of other ocular symptoms (sensitivity to light and poor vision) between the symptomatic and asymptomatic groups.

**Table 3:** Relationship between mucin levels and ocular symptoms frequency

Characteristic	No.	MUC1		MUC4		MUC16		MUC20		MUC5AC	
		M (IQR)	*P*-value^§^	M (IQR)	*P*-value^§^	M (IQR)	*P*-value^§^	M (IQR)	*P*-value^§^	M (IQR)	*P*-value^§^
Sensitivity to Light											
Symptomatic group	47	1.34 (2.98)	0.203	0.80 (1.94)	0.310	1.63 (2.17)	0.178	1.44 (2.03)	0.299	0.65 (1.60)	0.213
Asymptomatic group	32	0.67 (2.60)		1.14 (2.03)		0.84 (1.57)		0.71 (3.04)		1.64 (3.95)	
Foreign Body Sensation											
Symptomatic group	54	1.70 (3.39)	0.043*	1.01 (1.96)	0.886	1.68 (3.16)	0.017*	2.19 (2.77)	0.007*	0.93 (2.57)	0.948
Asymptomatic group	25	0.60 (0.94)		0.98 (1.96)		0.92 (1.13)		0.59 (0.56)		1.31(3.09)	
Painful or Sore Eyes											
Symptomatic group	29	1.73 (3.28)	0.202	1.05 (2.60)	0.830	1.63 (2.94)	0.158	2.24 (2.60)	0.031*	0.93 (2.24)	0.743
Asymptomatic group	50	0.74 (2.40)		0.98 (1.87)		1.03 (1.73)		0.83 (1.93)		1.04 (3.15)	
Blurred Vision											
Symptomatic group	56	1.50 (3.22)	0.011*	0.95 (2.06)	0.623	1.63 (2.49)	0.023*	2.03 (2.55)	0.009*	1.04 (3.03)	0.332
Asymptomatic group	23	0.35 (1.35)		1.14 (1.89)		0.84 (1.17)		0.49 (1.31)		0.54 (1.88)	
Poor Vision											
Symptomatic group	57	1.26 (2.82)	0.729	0.96 (1.93)	0.878	1.45 (2.29)	0.399	1.52 (2.36)	0.403	1.03 (3.18)	0.365
Asymptomatic group	22	0.88 (2.67)		1.13 (2.06)		1.03 (1.58)		0.81 (2.49)		0.72 (2.02)	

Symptomatic group: participants who answered *most of the time* or *all of the time* for each ocular symptom item. Asymptomatic group: participants who answered *half of the time* or *some of the time* or *none of the time* for each ocular symptom item. ^§^Mann-Whitney U test. M, median. IQR, interquartile range. *P < 0.05 between groups

### Relationship between mucin levels and parameters of DED

**Fig. 2:**
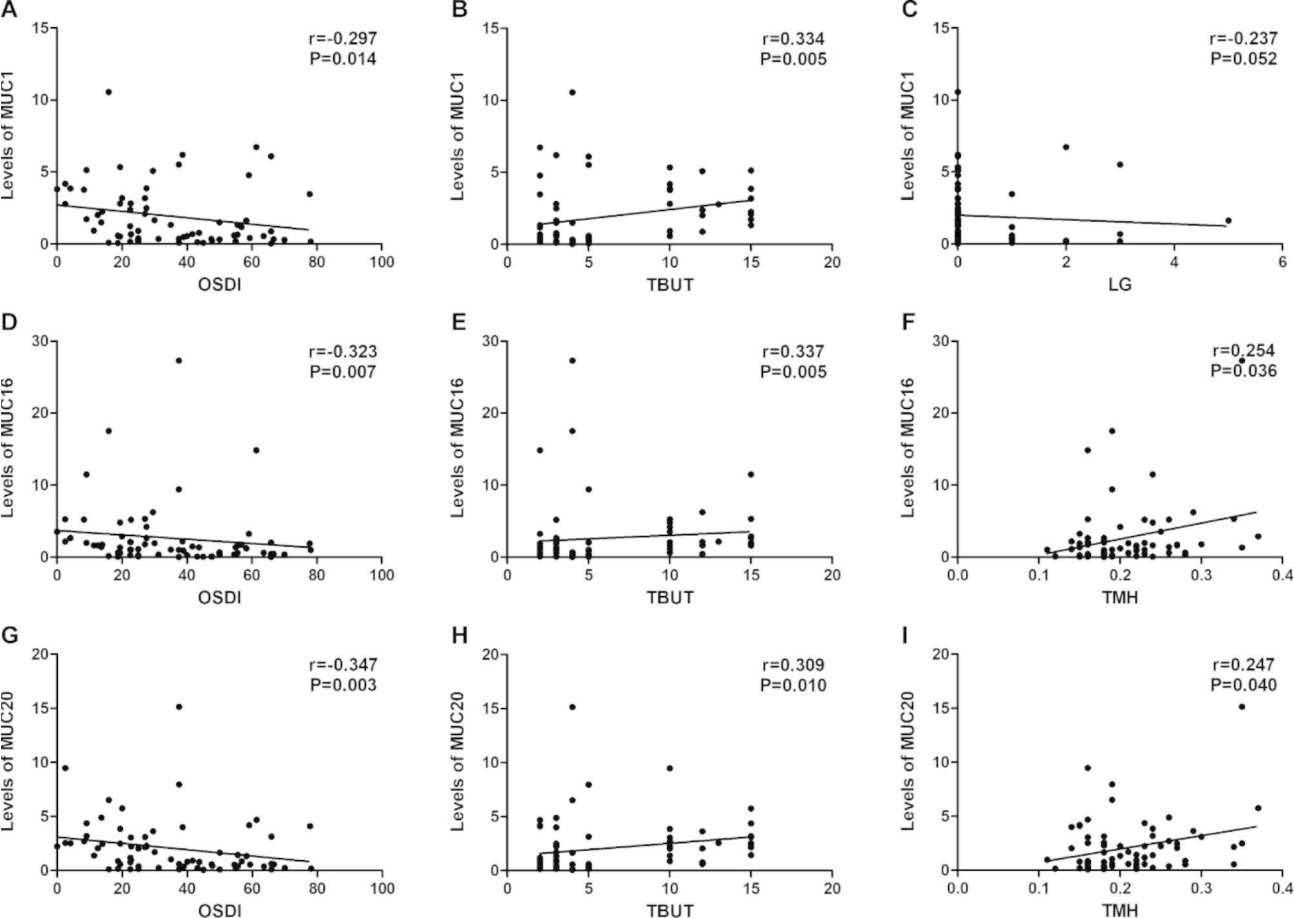
Correlation between mucin levels and parameters of DED in VDT users. Correlation of OSDI (**A**), TBUT (**B**) and LG (**C**) with levels of MUC1. (**D**), (**E**), and (**F**) showed the correlation of OSDI, TBUT, and TMH with MUC16 levels, respectively. (**G**), (**H**), and (**I**) showed the correlation of OSDI, TBUT, and TMH with levels of MUC20, respectively. r indicates the spearman correlation coefficient. *P* < 0.05 was considered statistically significant

As shown in Fig. 2, we found a significant negative relationship between the levels of MUC1, MUC16 and MUC20 and OSDI (r=-0.297, P = 0.014; r=-0.323, P = 0.007; r=-0.347, P = 0.003, respectively), a significant positive relationship between the levels of MUC1, MUC16 and MUC20 and TBUT (r = 0.334, P = 0.005; r = 0.337, P = 0.005; r = 0.309, P = 0.010, respectively), and a significant positive relationship between the levels of MUC16 and MUC20 and TMH (r = 0.254, P = 0.036; r = 0.247, P = 0.040, respectively). There were no significant correlations between MUC1, MUC16 and MUC20 levels and other DED parameters. In addition, no significant relationship was found between MUC4 and MUC5AC levels and all ocular surface parameters.

## Discussion

Data from this cross-sectional study showed a decrease in mRNA expression of ocular surface MAMs (MUC1, MUC16, and MUC20) in VDT users with DED. In addition, we investigated the relationship between the presence of symptoms and signs of DED and expression levels of five important mucins in VDT users.

The wet ocular surface includes the stratified squamous mucosal epithelia of the cornea/conjunctiva and the overlying tear film. The adjacent apical cells were sealed by forming tight junctions, which served as a paracellular barrier to prevent harmful substances from entering the ocular surface. In addition, the plasma membranes of apical cells develop folds called microplicae that protrude outward into the tear film and from which the mucosal glycocalyx is elaborated [19]. This glycocalyx is the boundary between the epithelium and tear film, and is mainly composed of MAMs, which serve as the backbone of the glycocalyx. The corneal and conjunctival epithelia express the following four MAMs: MUC1, MUC4, MUC16, and MUC20. The main functions of ocular surface MAMs include (1) surface protection against frictional stress (hydrodynamic lubrication), (2) apical cell surface barrier formation against pathogens and other environmental toxic agents (glycocalyx barrier), and (3) improvement of epithelium wettability (aqueous tear film anchorage) [12, 14]. Secreted mucins include soluble MUC7 and gel-forming MUC5AC. These mucins, particularly MUC5AC, assist in the removal of debris from the tear film and contribute to the hydrophilicity of the tear film. The model proposed by Dilly et al. revealed the role of mucins in tear film structure [20]. MAMs at the surface of upper epithelial cells form the deepest compartment of the tear film, while secreted mucins diffuse in the aqueous layer, from the glycocalyx to the lipid layer. Both MAMs and secreted mucins are vital for tear film quality and stability. Thus, alterations in the expression pattern of mucins may be related to the pathogenesis of DED.

This study enriched the alteration of mucins in different subtypes of DED, especially the changes of MAMs in VDT-associated DED, which has rarely been studied. In the present study, VDT users with DED showed decreased expression of conjunctival MUC1, MUC16, and MUC20 compared to VDT users without DED. Changes of MAMs in patients with DED have been observed previously but remain controversial. MAM expression and MUC1 immunoreactivity in the conjunctiva were reduced in patients with Sjögren’s syndrome (SS) [21, 22]. Similarly, Corrales et al. found that the mRNA expression of conjunctival MUC1 was significantly lower in patients with moderate to severe non-SS DED than in healthy subjects [23]. Additionally, the mRNA level of MUC16 was significantly lower in short tear breakup time and aqueous deficiency DED than that in normal controls [11]. However, Senchyna et al. found that SS patients displayed a significant increase in MUC1 and MUC16 mRNA expression compared with non-DED individuals [24, 25]. Furthermore, in postmenopausal women with non-SS DED, upregulation of conjunctival MUC1 and MUC16 mRNA or protein levels, or both, may be a compensatory response to irritation and inflammation associated with DED [26]. Altered expression of ocular mucins in DED varies considerably from study to study, likely attributed to differences in aetiology and disease severity of the included DED participants.

To our knowledge, this is the first study to assess the alteration of MUC20 at the ocular surface in patients with DED. Microarray analysis of CIC indicates that MUC20, a relatively new member of the MAMs family, is the most highly expressed mucin gene in human conjunctiva [27]. In 2014, Woodward et al. reported that MUC20 has a unique localization pattern that differs from other MAMs at the ocular surface. It was predominant in the intermediate cell layers of the conjunctival and corneal stratified epithelia rather than the apical layers [15]. In addition, MUC20 appears to be a non-secreted and non-shed MAM which is not found in tears. Data obtained in our study of VDT users showed that DED patients displayed lower conjunctival MUC20 mRNA expression than the control participants. Thus, we hypothesized that alterations in MUC20 play a critical role in the development of DED. Overall, in our study, patients with VDT-associated DED showed a unique pathogenic pattern dominated by MAMs damage.

Our data suggest that the expression of MUC5AC at the surface of the eye is minimally affected by the VDT-associated DED. Although there was a tendency toward a reduction in the expression of MUC5AC mRNA in VDT users with DED, the significance was not statistically significant when compared with the control group. Similarly, Uchino et al. found no significant differences in conjunctival MUC5AC expression in VDT users with DED compared with those without DED [17]. In a study by Gipson [26], the amount of goblet cell-derived MUC5AC in conjunctival cells or tear samples did not differ significantly between postmenopausal women with and without a history of DED. These data differ from those of Argüeso et al. and Zhao et al., who found decreased MUC5AC expression or tear protein levels in patients with SS-DED and non-SS DED compared with normal subjects [28, 29]. The discrepancy between these studies may be due to differences in the severity of DED or the measurement protocols. Further studies are required to determine whether the mRNA level of MUC5AC could serve as a marker to characterize dry eye severity and progression in VDT users.

Interestingly, we found that the levels of MUC1, MUC16, and MUC20 in VDT users were correlated with several subjective symptoms and objective ocular surface parameters (mainly TBUT and TMH). The function of MAMs is to maintain the wet ocular surface and stabilize the tear film, so the loss of MAMs may lead to tear film instability and reduced tear volume, which in turn causes shortened TBUT and reduced TMH, respectively [30]. The results of therapeutic studies have also shown that mucin secretagogue (3% diquafosol tetrasodium) treatment increases TBUT in patients with DED [31]. Considering that MAMs contribute to the increased wettability of the ocular surface, we speculate that deficiency of MUC1, MUC16 and MUC20 causes uncomfortable symptoms of DED by affecting tear film stability and tear volume.

Currently, several drugs specifically target mucin deficiency in DED by increasing the expression and secretion of mucin at the surface of the eye, including diquafosol, a purinergic P2Y2 receptor agonist, and rebamipide, an amino acid derivative of 2-(1 H)-quinolinone. A clinical trial showed that both diquafosol and rebamipide are effective in the treatment of DED in office workers [32]. Furthermore, recent research has suggested that a new ophthalmic pharmaceutical formulation, topical sulglycotide, enhances ocular MAMs including MUC1, MUC4, and MUC16, as well as MUC5AC secretion in DED [33]. These studies also confirmed the critical role of mucin alterations in the pathogenesis of VDT-associated DED from a therapeutic perspective.

A limitation of our study is that we only examined the mRNA expression of conjunctival mucins in VDT users rather than protein levels, which also needs to be explored in the future. In addition, mucin levels based on CIC samples may not be completely representative of those in all conjunctiva. Considering the convenience of collecting samples and patient compliance, we only collected CIC samples in the temporal location of the conjunctiva in all participants to analyse mucin expression.

## Conclusion

Conjunctival MUC1, MUC16, and MUC20 expressions were lower in VDT users with DED than in those without DED. Furthermore, VDT users with increased frequency of ocular discomfort and poor tear film stability have significantly reduced conjunctival MAMs levels. Understanding mucin alterations associated with VDT-associated DED is crucial for elucidating its progression mechanisms and finding effective treatment strategies.

## Data Availability

All data generated or analysed during this study are included in this published. The relevant raw data will be freely available from the corresponding author upon request.
